# Supplementary material for: Pore cross-talk in colloidal filtration
Source: Sci Rep. 2018 Aug 20;8:12460. doi: 10.1038/s41598-018-30389-7 (PMC6102238; doi:10.1038/s41598-018-30389-7)
Supplement: Supplementary file 1 — Supplementary materials [file 41598_2018_30389_MOESM1_ESM.pdf]

Pore cross-talk in colloidal filtration

—

Supplementary materials

Olivier Liot, Akash Singh, Patrice Bacchin, Paul Duru, Jeffrey Morris, and Pierre Joseph

LAAS-CNRS, 7 avenue du colonel Roche, 31400 Toulouse, France

(Dated: April 12, 2018)

1. GROWTH RATE ASSESSMENT

The clog growth rate is assessed in the linear part of the quick growth zone of the clog area vs time evolution. The start point (t_{start}) of this part systematically corresponds to the sudden transition from the slow to the quick growth rate part. It can be manually determined without ambiguity. The end point (t_{end}) is also determined manually. The resulting curve is filtered by convolution with a Gaussian filter [1]. Then an affine fit is performed to extract the clog growth rate (in $\mu m^2.s^{-1}$).

If the second point used to fix the domain where clog growth rate is computed is chosen in the zone where the clog area vs time evolution is clearly linear, there is no noticeable effect on the growth rate. Figure 1 (left) shows one clog area vs time evolution. Figure 1 (right) shows the computed clog growth rate when the point at the end of the domain is changed. One can observe that the dispersion does not exceed 10%, which remains in the statistical uncertainty over the different experiments.

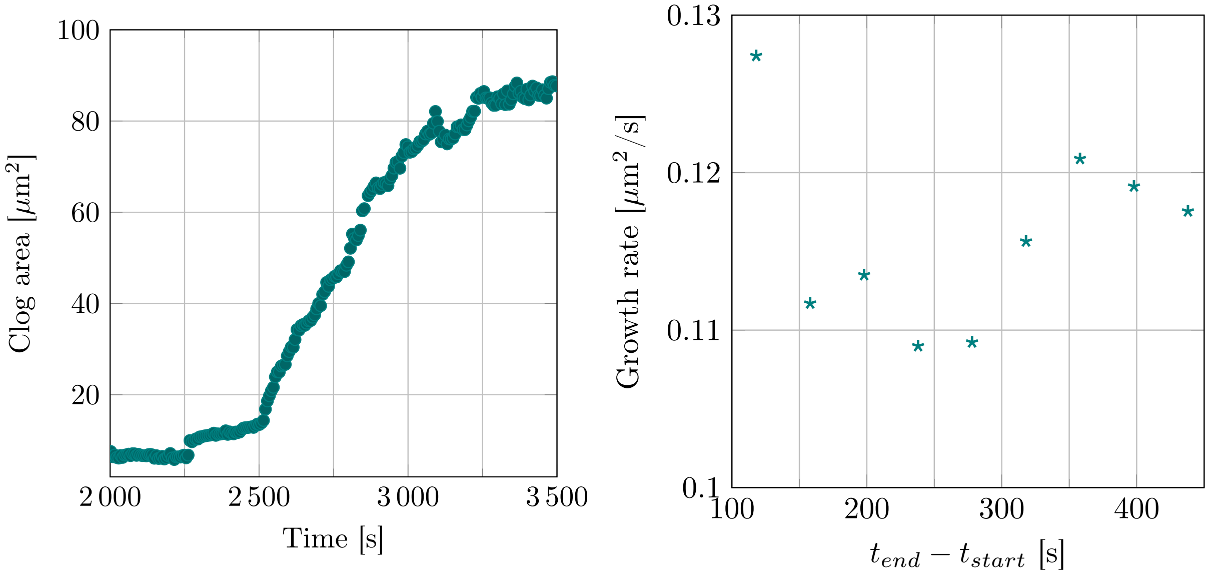

FIG. 1. (left) Clog area evolution vs time. (right) Computed growth rate depending on the difference between chosen end time and start time.

2. CLOGGING ONSET PREFERENTIALLY TRIGGERED BY PROXIMITY OF NEW SATURATED PORE

When a pore is saturated, the first neighbouring pores, if it/they is/are free, receive a more important concentration of particles. Let us consider the probability of the next saturated pore to be at a distance Δx from the newly one. If there were no redistribution, we should expect a random process. We can observe in figure 2 that this probability departs from a stochastic process only for one inter-pore distance. It is a signature that the saturated pore has an influence only on the first neighbouring free pores (both sides). If the first free clog is at more than one inter-pore distance, its clogging onset has already been influenced by its own first saturated neighbouring pore(s).

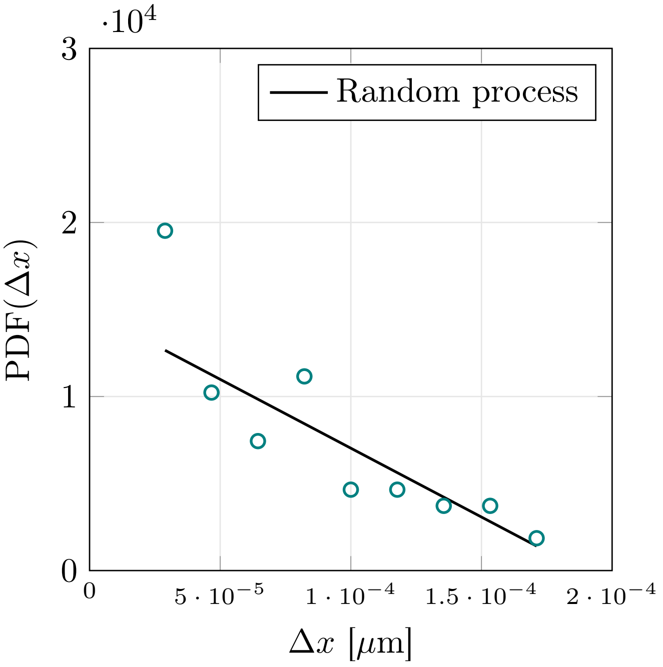

FIG. 2. Probability of the next saturated pore to be at a distance Δx from the newly one.

3. COMPUTATION OF $\langle k \rangle_N$

We have to compute the average number of successive saturated pores neighbouring free pores $\langle k \rangle_N$. We consider a 1-D array of N_{tot} pores. The total number of saturated/free pores configurations reaches $2^{N_{tot}}$. For a given N_{tot} we construct the tree diagram when we draw

successively all the pores and observe if they are free or saturated. At each step, there are two possible events: either the drawn pore is saturated (event S), or it is free (event F). Figure 3 shows the diagram tree for $N_{tot} = 2$.

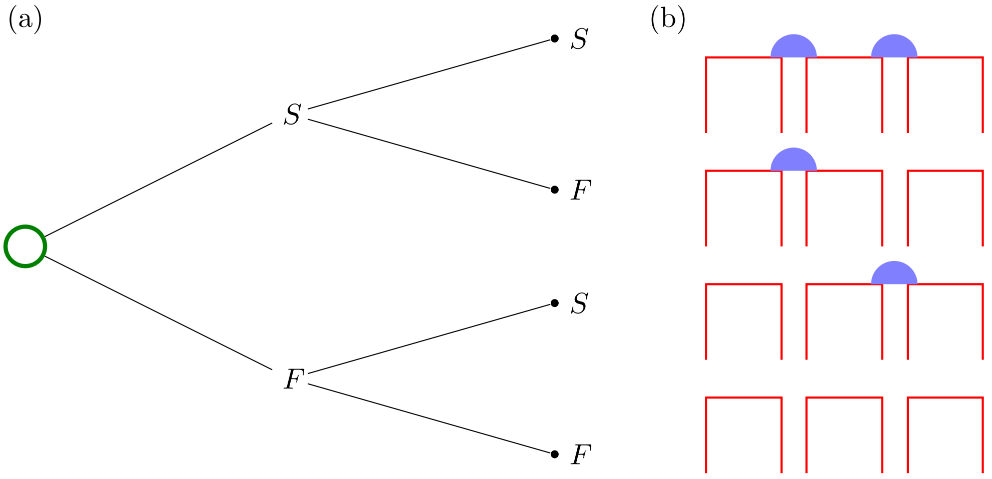

FIG. 3. (a) Tree diagram representing the possible configurations of $N_{tot} = 2$ pores. (b) Sketch of the corresponding configurations.

This diagram tree is numerically built to estimate, for each path in the set of paths $\mathcal{P}$ (and so for each configuration), the average number of successive saturated neighbours for the free pores. Then N is fixed and only the possible paths $\mathcal{P}_N$, corresponding to paths with N saturated pores, are kept (for example if $N = N_{tot} - 1$, only N_{tot} paths are possible). All these paths have the same probability to occur. We count for each path the mean number of successive saturated pores neighbouring the free pores. Averaging on all paths in $\mathcal{P}_N$ leads to the mean value $\langle k \rangle_N$. The same operation is repeated for each $N \in [0, N_{tot} - 1]$. The figure 4 shows $\langle k \rangle_N$ as a function of N for $N_{tot} = 10$. As expected it is increasing, with a maximal value of 9 for $N = 9$, as it can be intuitively expected.

[1] G. A. Voth, A. La Porta, A. M. Crawford, J. Alexander, and E. Bodenschatz, Journal of Fluid Mechanics **469** (2002), 10.1017/S0022112002001842.

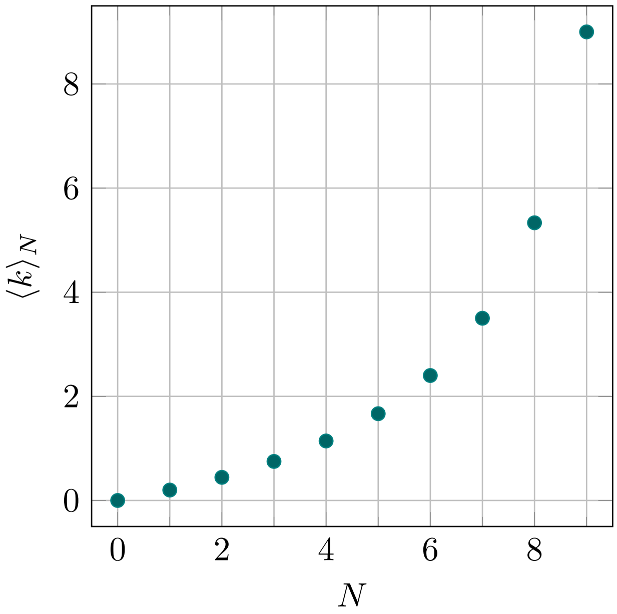

FIG. 4. Average of the number of successive saturated pores neighbouring free pores versus N for $N_{tot} = 10$.
